# Enhanced Bioavailability of Boswellic Acid by *Piper longum*: A Computational and Pharmacokinetic Study

**DOI:** 10.3389/fphar.2020.551911

**Published:** 2020-12-15

**Authors:** K. Reeta Vijayarani, Manoj Govindarajulu, Sindhu Ramesh, Mansour Alturki, Mohammed Majrashi, Ayaka Fujihashi, Mohammed Almaghrabi, N. Kirubakaran, Jun Ren, R. Jayachandra Babu, Forrest Smith, Timothy Moore, Muralikrishnan Dhanasekaran

**Affiliations:** ^1^ Department of Pharmaceutics, Periyar College of Pharmaceutical Sciences, Tiruchirappalli, India; ^2^ Department of Drug Discovery and Development, Harrison School of Pharmacy, Auburn University, Auburn, AL, United States; ^3^ Department of Pharmaceutical Chemistry, College of Clinical Pharmacy, Imam Abdulrahman Bin Faisal University, Dammam, Saudi Arabia; ^4^ Department of Pharmacology, Faculty of Medicine, University of Jeddah, Jeddah, Saudi Arabia; ^5^ Department of Medicinal Chemistry, College of Pharmacy, Taibah University, Medina, Saudi Arabia; ^6^ School of Pharmacy, University of Wyoming College of Health Sciences, Laramie, WY, United States

**Keywords:** anti-inflammatory, boswellic acid, bioavailability, computational analysis, natural products, *Piper longum* extract

## Abstract

Chronic inflammation is a key culprit factor in the onset and progression of several diseases. Novel and pharmacologically effective therapeutic approaches are needed for new treatment remedy or improved pharmacokinetics and pharmacodynamics for existing synthetic drugs, in particular natural products. Boswellic acids are well-known natural products, with capacity to effectively retard inflammation without severe adverse effects. However, the therapeutic use of Boswellic acids are greatly hindered by its poor pharmacokinetic properties. Co-administration strategies that facilitate the oral absorption and distribution of Boswellic acids should lead to a safe and more effective use of this product prophylactically and therapeutically in inflammatory disorders. In this study, we examined the effect of *Piper longum* extract on the absorption and bioavailability of Boswellic acid in rabbits. In addition, we further explored computational pharmacodynamic interactions between *Piper longum* and Boswellic acid. *Piper longum* extract at 2.5 and 10 mg/kg, increased the bioavailability of Boswellic acid (*p* < 0.05). Based on our drug-based computational modeling, cytochrome P450 (CYP450)-mediated mechanism was involved in increased bioavailability. These findings confirmed that *Piper longum* with Boswellic acid may be administered orally together for effective therapeutic efficacy. Thus, our studies support the application of *Piper longum* with Boswellic acid as a novel therapeutic avenue in diseases associated with inflammation.

## Introduction

Inflammation response occurs as a result of increased pro-inflammatory and/or decreased anti-inflammatory mediators, promoting acute or chronic pathological conditions (Ren and Wu, 2006; Tan et al., 2019). Chronic inflammation has a central and inciting role in the development of a wide variety of disorders. Conventional anti-inflammatory agents administered for long period are associated with several adverse events including gastrointestinal and cardiovascular toxicity. Hence, there is a growing interest in utilizing natural products as a potential anti-inflammatory agent.

**FIGURE 1:**
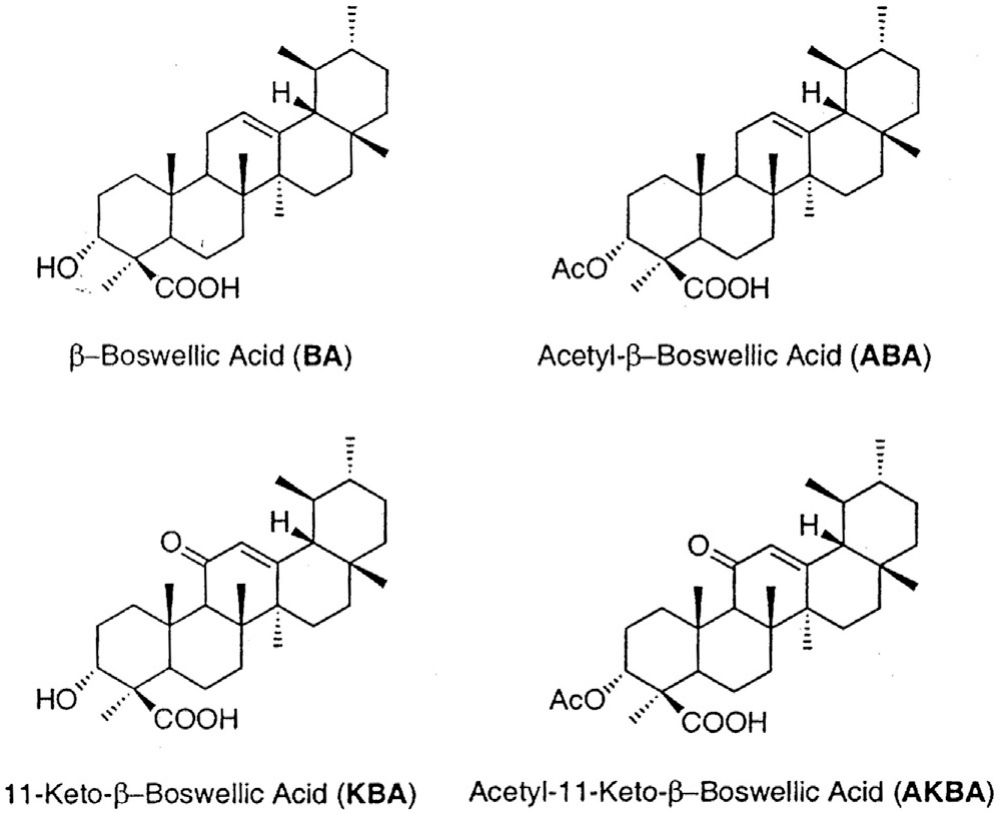
**(A)** β-Boswellic Acid (BA). **(B)** Acetyl β-Boswellic Acid (ABA). **(C)** 11-Keto-β-Boswellic Acid (KBA). **(D)** Acetyl-11-Keto-β-Boswellic Acid (AKBA).

Boswellic acids have been known to be promising candidates in the treatment of inflammatory diseases. The gum resin of *Boswellia serrata* (Indian frankincense, Sali Guggal) is native to the Punjab region of Asia. Similar to *Mucuna pruriens* (Manyam et al., 2004a; Manyam et al., 2004b), *Centella asiatica* (Dhanasekaran et al., 2007) and *Bacopa monniera* (Dhanasekaran et al., 2009), *Boswellia serrata* has also been traditionally used in Ayurvedic medicine. The bark of *Boswellia serrata* contains essential oils, polysaccharides, and various resins. However, the most notable component from the bark belongs to boswellic acids. Boswellic acids are part of a larger group of compounds commonly known as triterpenes, which exhibit anti-inflammatory activities mainly by modulating nuclear factor-kappaB (NF-κB) and STAT3 transcription factors (Ammon, 2016). In addition, 11-keto-β-boswellic acid (KBA) and 3-O-acetyl-11-keto-β-boswellic acid (AKBA) (Figure 1) noncompetitively inhibit 5-lipoxygenase, serine protease-cathepsin G and microsomal prostaglandin E synthase (Bertocchi et al., 2018). Conversely, pharmacokinetic studies of boswellic acids only revealed poor bioavailability, especially with regards to the two boswellic acids contents namely KBA and AKBA. Several studies have shown that despite the use of high doses of boswellic acid derivatives in oral administration, little or none detectable levels of these active derivatives can be found in human biological blood or fluids (Sterk et al., 2004; Krüger et al., 2008; Sharma et al., 2010)^.^ The current scientific hypothesis is that poor absorption and high metabolism of boswellic acids may be responsible for the low bioavailability. The poor absorption has been attributed to their low water solubility and hydrophobicity after oral administration. In order to increase the poor absorption due to low solubility, various approaches have been investigated. A study by Meins et al., investigated the effect of AQUANOVA micellation to improve bioavailability in rats and found that micellar solubilization improves bioavailability of poorly soluble substances such as boswellic acids (Meins et al., 2018). Similarly, a soy lecithin formulation of boswellic extract gum resin improved systemic availability and tissue distribution (Husch et al., 2013). The current study focused on improving the pharmacokinetic properties of boswellic acids by co-administration of piperine.

*Piper longum* has recently gained scientific interest for one of its main and abundant alkaloids called piperine. Many studies have demonstrated that piperine affects drug metabolism (Bhardwaj et al., 2002; Krüger et al., 2008; Singh and Duggal, 2009; Cui et al., 2016) and has shown to increase the bioavailability of many therapeutic substances such as curcumin, epigallocatechin-3-gallate, coenzyme Q10 and β-carotene (Kesarwani and Gupta, 2013). Hence, in this study we investigated the effects of *Piper longum* extract on the bioavailability of boswellic acids. Furthermore, novel computational analysis was performed to validate the potentiating effects of *Piper longum* on boswellic acid.

## Materials and Methods

Boswellic acid fraction from the gum resin of *Boswellia serrata* and the extract of dried fruits of *Piper longum* were supplied by T.T.K Pharma (P) Ltd., Chennai. Dichloromethane was purchased from Sigma-Aldrich, Bengaluru, Methanol, other chemicals and solvents (HPLC grade) were procured from Hi Media, Mumbai.

Isolation procedure of β-boswellic acid was performed by the following procedure. Olibanum (1 kg) was divided into two portions and dissolved in ether (1 L) by placing it in a shaker for 14 h. Following filtration of the ethereal extract it, was treated with barium hydroxide (30 g). The barium salt of the crude boswellic acid was immediately precipitated, filtered off, washed with ether, and dried to a pale-yellow solid (330 g). The barium salt was then boiled for 4 h with acetic anhydride (500 g) containing a small amount of pyridine. On cooling the mixed anhydride of crude boswellic acid acetate was crystallized and separated. The mixed anhydride was dissolved in chloroform and heated under reflux with methanol for 1 h. The crystallized product form chloroform-methanol gave crude β-boswellic acid acetate crystals with melting point 262–269°C (Yield 53 g as rhombic crystals). Further crystallization from chloroform-methanol provided a mixture of β-boswellic acid acetate (3α-acetooxyurs-12-en-24-oic acid) and ca-30% of 3α-acetoxyursa-9 (11): 12–dien-24-oicacid. This mixture had a melting point 273–276°C and an absorption maximum at 2810A⁰(ε3420). From different preparations of β-boswellic acid acetate, samples containing varying quantities of the diene as impurity were obtained. Samples of methyl β-boswellate acetate contaminated with the corresponding dehydro compound were obtained by methylation of ethereal solutions of free acid with diazo methane.

For further purification, mixture of β-boswellic acid acetate and the corresponding diene were treated with lithium in liquid ammonia in the presence of ethanol: Mixture (ε3420 at 2810A⁰, corresponding to 30% diene content) (500 mg) in ether (25 ml) dioxin (25 ml) and ammonia (ca.50 ml) was treated with lithium (3 kg). The mixture was stirred for 1 h for dissolving the lithium. Ethanol was added dropwise until the solution became colorless. The solution was then added to water, acidified with hydrochloric acid and extracted with ether. The product was reacetylated with acetic anhydride-pyridine to give β-boswellic acid acetate, melting point-275–278°C (from methanol). After a second reduction with lithium and ammonia as above, the product showed slight absorption at 281OA⁰(5–10) indicating that the last traces of diene are not easily removed. Hydrolysis of β-boswellic acid acetate with 10% methanolic potassium hydroxide provided β-boswellic acid (3α-hydroxyurs-12-en-24-oic acid) as rhomb melting point (212–215°C). Most of the diene impurity was eliminated by treatment of β-boswellic acid with lithium in ammonia in the presence of ethanol, a product containing 0.5% of diene impurity being thus obtained: their rotations were lower than any described in the literature.

High performance liquid chromatography (HPLC, Shimadzu) using ultraviolet (UV) detector (Shimadzu UV-Visible variable wavelength detector SPD-10A) with Shimadzu CRA chromatopac was used for the estimation of β-boswellic acid. The other parameters used for the detection β-boswellic acid were the mobile phase (methanol-70% and water-30%) with the flow rate (0.5 ml/min) and a reverse phase C18 ODS column. The wavelength used to detect β-boswellic acid was 249 nm. Initially, standard curve for β-boswellic acid was obtained by preparing a stock solution and diluting it to various concentrations (0, 0.125, 0.25, 0.5,1.0 μg/ml) in methanol. The minimum quantity that can be detected was 0.05 μg/ml. The method was sensitive and linear with an R^2^ for the standard curve injections was 0.997. Serum obtained from animals was subjected to extraction with dichloromethane and evaporated in water bath. The residue was dissolved in mobile phase, followed by filtration through the membrane filter (0.45 µ) and the filtrates were injected into the HPLC using 20 µl loop. The β-boswellic acid contents in serum were calculated from the standard curve obtained. For authentication purpose, β-boswellic acid (standard various concentrations) was also injected after the analysis of samples. To further validate the data obtained, serum from control animals were spiked with known amount of β-boswellic acid (0.125, 0.5 and 1 μg/ml) and analyzed in HPLC-UV. The retention time of the peaks were similar as compared to the unspiked sample. The amount of boswellic acid recovered was 95.54 + 0.05%. In addition, other types/derivatives of boswellic acid was not significantly detected with these HPLC parameters.

### Preparation of Animals for the Pharmacokinetic Study

**FIGURE 2:**
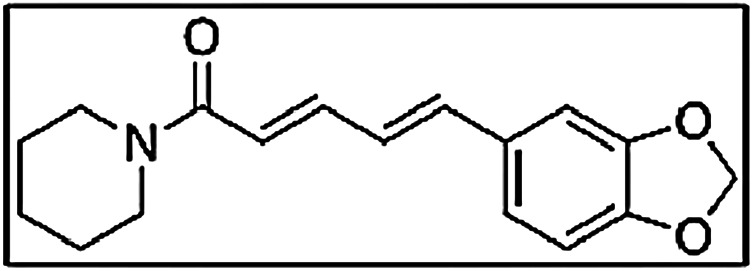
Structure of piperine.

The animal study was carried out with prior approval from the institution’s animal ethical committee (PCP/IAEC/009/2008). The rabbits were treated after an acclimatization period of 15 days to the laboratory environment. Eighteen healthy male rabbits (aged 6 months) weighing around 1.5 kg were selected and divided into three groups. The first group served as a control was treated with 20 mg/kg of β-boswellic acid fraction of *Boswellia serrata*. Based on the existing, the dose was obtained from previous study conducted by Kumar et al., and Sharma et al. These studies investigated the anti-inflammatory effects of Boswellic acid, and the doses used were in the range 0–100 mg/kg. Since, we are studying the effect of *Piper longum* on the bioavailability of β-boswellic acid, we performed the study with 20 mg/kg (Sharma et al., 1989; Kumar et al., 2019; Ammon, 2016). Previous studies have indicated that the effective bioenhancing dose of piperine (*Piper longum extract*) for drugs varies and that a dose of approximately 10% (wt/wt) of the active drug or a daily dose of 10–20 mg/day to be regarded as an appropriate bioenhancing dose for majority of the drugs (Wadhwa et al., 2014). We did not quantitatively measure the piperine quantity and hence based on the literature studies, we utilized two doses of *Piper longum* extract (2.5 and 10 mg) to evaluate the bioavailability of β-boswellic acid. The second group was treated with 20 mg β-boswellic acid fraction of *Boswellia serrata* and 2.5 mg of *Piper longum extract*. The third group was treated with 20 mg of β-boswellic acid fraction of *Boswellia serrrata* and 10 mg of *Piper longum extract* (Figure 2). The medicaments were blended uniformly and filled in size 0 capsules and were given orally to rabbits with the help of a capsule holder.

### Blood Sampling and Drug Analysis

Blood samples withdrawn from the marginal ear vein of the rabbits at periodic time intervals were centrifuged and the serum was collected to measure β-boswellic acid using HPLC-UV.

### Computational Analysis

Drug molecules with favorable absorption, distribution, metabolism, and elimination (ADME) properties have been identified as the primary indicators of successful candidate molecules in drug discovery and development. In this study, QikProp filter from Schrödinger was used to calculate several pharmacokinetic and pharmacodynamic properties of piperine, AKBA, and KBA. The Qikprop set of descriptors (SASA, FOSA, FISA, PISA, #metabolites, CNS distribution, QPlog BB, Donor HB, Accept HB, logP, % human oral absorption, and Rule of 5) were selected to describe permeability, metabolism and activity. Schrödinger release 2020: QikProp, Schrödinger, LLC, New York, NY software was used in the computational analysis.

### Statistical Analysis

Prism 5 software (La Jolla, CA, United States) was used for statistical analysis. Statistical analyses were accomplished using one-way analysis of variance (ANOVA) followed by Dunnet's multiple comparisons test (*p* < 0.05) and was determined to be statistically significant. Results were expressed as mean ± SEM.

## Results

**FIGURE 3:**
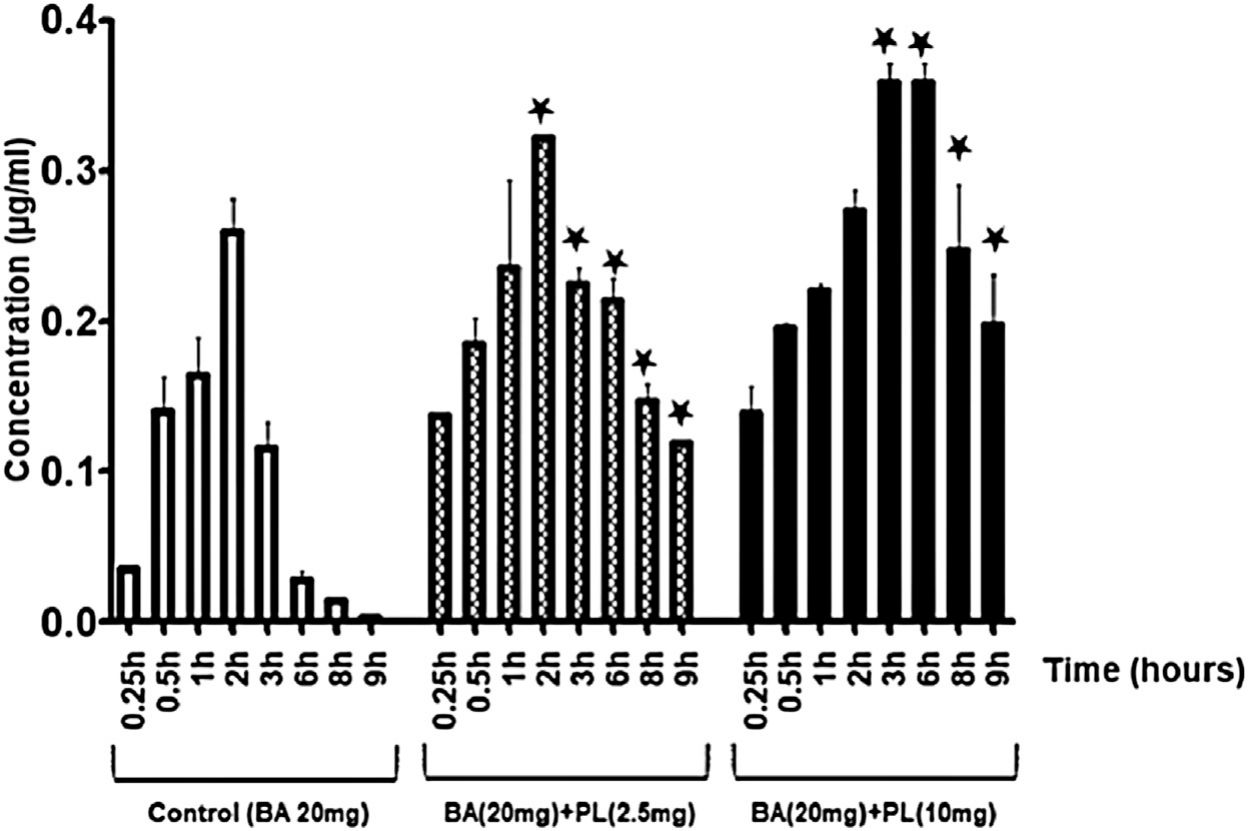
*Piper longum* extract (2.5 and 10 mg/kg) increases the serum concentration of β-boswellic acid in rabbits (*p < 0.05). BA-β-boswellic acid (20 mg/kg).

The pharmacokinetic data in rabbits show the highest serum concentration (Tmax) of β-boswellic acid peaks at 2 h and then decreases subsequently with complete elimination at 9 hours (Figure 3). However, *Piper longum* increased the serum concentration of β-boswellic acid significantly in rabbits and the effects were noticed up to 9 h (*p* < 0.05). The pharmacokinetic parameters of *Piper longum* extract co-administered with β-boswellic acid are outlined in Table 1. Specifically, the maximum serum concentration (Cmax) of β-boswellic acid achieved by 0, 2.5 and 10 mg/kg *Piper longum* extract co-administration was 0.2599, 0.3214 and 0.3589 μg/ml respectively (Table 1). Similarly, the area under serum concentration versus time curves were 0.7736, 1.18828, and 2.2045 µg/ml/h respectively (Table 1). The data suggests that the bioavailability of β-boswellic acid was significantly increased by *Piper longum* extract.

**TABLE 1:** Pharmacokinetic data of control, Test I and Test II groups.

Pharmacokinetic Parameters	Control	Test I	Test II
Dose administered (in mg)	20 mg of β-boswellic acid (BA)	20 mg of BA + 2.5 mg of adjuvant (PL)	20 mg of BA + 10 mg of adjuvant (PL)
AUC_(0-t)_ (µg/ml/h)	0.7656 ± 0.0228	1.8874 ± 0.1091	2.2138 ± 0.1145
AUC_(0-inf)_ (µg/ml/h)	0.7716 ± 0.0221	2.6604 ± 0.1018	2.8547 ± 0.2512
Relative bioavailability	—	2.424	2.8496
Cmax (µg/ml)	0.2599 ± 0.0213	0.3214 ± 0.0027	0.3589 ± 0.0120
Tmax (h)	2 ± 0.18	2 ± 0.22	3 ± 0.26
Ke (h^−1)^	0.47 ± 0.0843	0.1888 ± 0.0365	0.1106 ± 0.0505
(t^1/2^)_e_ (h^−1)^	1.46 ± 0.28	3.67 ± 0.61	6.26 ± 0.87
Ka (h^−1)^	0.6168 ± 0.16	0.740 ± 0.22	0.6112 ± 0.17
(t^1/2^)_a_ (h^−1)^	1.12 ± 0.24	0.93 ± 0.11	1.13 ± 0.3
Apparent volume of distribution (V_d_/F) (L)	96 ± 14	43.24 ± 7	67.5 ± 11

QikProp Assessment: Pharmacokinetic and pharmacodynamic properties of piperine, AKBA and KBA were analyzed using QikProp, Schrödinger software. Solvent accessible surface area (SASA) of a molecule is its surface area that is in contact with the solvent in the biological system.

Lower scores of SASA mean that more of the molecule is interacting with a biomolecule like a protein or a membrane and most of it will likely remain in the unionized form; hence, higher absorption and bioavailability. On the other hand, higher SASA scores indicate that more of the molecule is interacting with the solvent such as the aqueous medium of the stomach (stomach acid), and most of it will likely stay in the ionized state; thus, lower absorption and bioavailability. In Table 2, piperine shows a lower SASA value compared to other boswellic acid fractions (β-boswellic acid, KBA and AKBA) which are consistent with what has been noted elsewhere for piperine’s favorable bioavailability profile. Other parameters like hydrophobic components of the SASA (FOSA), hydrophilic components of the SASA (FISA), and π (carbon and attached hydrogen) components of the SASA (PISA) values for piperine, β-boswellic acid, AKBA, and KBA are all within the acceptable range (Dudhatra et al., 2012).

**TABLE 2:** SASA, FOSA, FISA, PISA #metab, CNS, and QPlog BB calculations of the molecules.

Compound	SASA(Å)	FOSA(Å)	FISA (Å)	PISA (Å)	#metab	CNS	QPlog BB
Piperine	541.54	317.96	43.2	180.37	0	0	−0.12
KBA	680.37	541.52	125.49	13.35	3	−1	−0.63
AKBA	734.77	586.51	134.9	13.35	2	−1	−0.76
Beta-boswellic acid	648.618	557.65	90.9	0.064	3	−1	−0.31
Aspirin	375.34	94.41	123.14	157.77	0	−1	−0.54
Hybrid-1	1025.72	818.96	81.1	125.65	5	−1	−0.73
Hybrid-2	1063.65	847.92	81.96	133.76	5	−1	−0.79
Hybrid-3	1113.03	897.36	85.67	130.01	4	−1	−0.85
Hybrid-4	1061.11	842.73	97.81	120.56	4	−1	−0.92

The permissible ranges are as follows: Total solvent accessible surface area (SASA) in square angstroms using a probe with a 1.4 Å Radius (300–1000), FOSA: Hydrophobic components of the SASA (0.0–750.0), FISA: Hydrophilic components of the SASA (7.0–330.0), PISA: π (carbon and attached hydrogen) components of the SASA (0.0–450.0), #metab: Number of likely metabolic reactions (1–8) CNS: −2 (inactive) to +2 (active), QPlog BB: (−3.0 to −1.2) polar compounds have large negative values. SASA: Solvent Accessible Surface.

In terms of metabolic reactions predictions (#metab), β-boswellic acid and KBA have the highest number of metabolic reactions, followed by AKBA and piperine, thus it validates the parameters that was observed experimentally (Krüger et al., 2008; Cui et al., 2016). In addition, β-boswellic acid, KBA, and AKBA show larger negative QPlog BB values compared to piperine, which indicate limited accessibility into the blood brain barrier by passive diffusion and will likely be concentrated in the peripheral system. For CNS activity, β-boswellic acid, KBA, and AKBA display lower activity compared to piperine, this substantiates the QPlog BB prediction.

Other parameters, which include, % Human oral absorption and Lipinski’s rule of five are within the acceptable range of drug bioavailability (Table 3).

**TABLE 3:** Calculated ADME properties of the molecules.

Compound	Molecular weight	HB Donor	HB Accept	cLogP	% Human oral absorption	Rule of 5
Piperine	285.34	0	4.5	3.1	100	0
KBA	470.69	1	4.7	5.6	86.7	1
AKBA	512.72	1	6	5.8	73.05	2
Beta-boswellic acid	456.70	1	2.7	6.2	96.2	1
Aspirin	180.16	1	4.5	1.1	73.8	0
Hybrid-1	756.03	0	9.2	7.7	100	2
Hybrid-2	756.03	0	9.2	8.01	100	2
Hybrid-3	798.07	0	10.5	8.1	100	2
Hybrid-4	798.07	0	10.5	7.6	100	2

The permissible ranges are as follows: Mol weight: (130–725), Hydrogen bond (HB) Donor: (0.0–6.0), Hydrogen bond (HB) Acceptor: (2.0–20.0), cLogP: (−2.0 to 6.5), % Human oral absorption: >80% high, <25% low, Rule of five (maximum 4).

**FIGURE 4:**
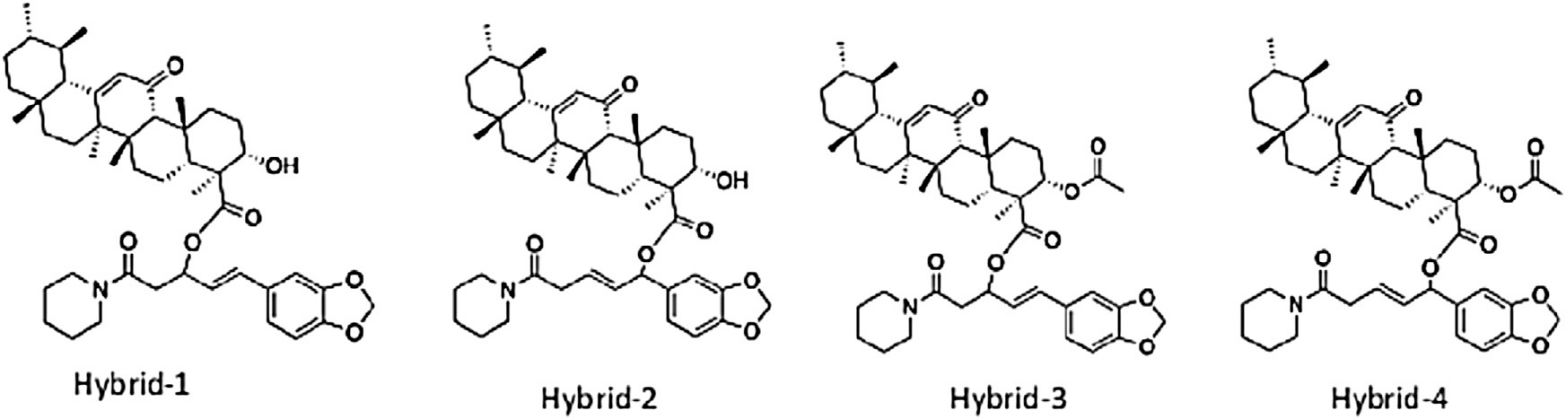
Rationally designed novel hybrid molecules of piperine and boswellic acids that could be synthesized to enhance pharmacokinetic and pharmacodynamics effects to reduce inflammation. **(A)** Hybrid-1. **(B)** Hybrid-2. **(C)** Hybrid-3. **(D)** Hybrid-4.

Utilizing rational drug design strategies, such as “hybridization” or “mix-n-match” of piperine with β-boswellic acid, AKBA or KBA, our results shed more lights on the mechanism of piperine enhancement of boswellic acid fractions. Considering this, four hybrid compounds were designed (Figure 4), by covalently linking the accessible carboxylic acid functionality of AKBA, and KBA to piperine by utilizing one to four and one to six conjugate addition reactions (Table 4). The QikProp prediction results of the designed hybrids show that the physiochemical properties of these compounds do not fall within the Lipinski’s rule of 5 (Ro5) space (Tables 2, 3) but fall within a recently identified chemical space called beyond the rule of 5 or simply (bRo5). The AbbVie multiparametric score (AB-MPS) prediction tool was used to predict the likelihood of a compound bRo5 to have an acceptable oral absorption. AB-MPS = Abs (cLogD-3)+NAR + NRB, where cLogD = is the calculated distribution coefficient widely used to calculate the lipophilicity of ionizable compounds at physiological pH 7.4, NAR = is the number of aromatic rings, NRB = is the number of rotatable bonds. Compounds with ≤14 scores are considered to have a higher likelihood for an acceptable oral absorption. An interesting aspect of the hybrid compounds that they showed scores that are less than 14 (Table 4). It should be noted that this tool is useful to rule out poor oral compounds bRo5 range in early drug discovery process.

**TABLE 4:** Calculated AB-MPS properties of the hybrid molecules.

Compound	LogD	NAR	NRB	AB-MPS^a^
Hybrid-1	8.39	1	7	13.39
Hybrid-2	8.51	1	7	13.51
Hybrid-3	8.83	1	7	13.83
Hybrid-4	8.95	1	7	13.95

cLogD is the calculated distribution coefficient to calculate the lipophilicity of ionizable compounds at physiological pH 7.4; NAR is the number of aromatic rings; NRB is the number of rotatable bonds; AB-MPS is the AbbVie multiparametric score.

a ≤14 scores are considered to have a higher likelihood for an acceptable oral absorption.

## Discussion

Combination of multiple drugs/botanicals in a single formulation (oral, dermal, parenteral, rectal) often results in much augmented bioavailability, leading to improved pharmacokinetic and pharmacodynamic properties, decreased adverse drug effects, and minimal hypersensitivity reactions, resulting in a novel and effective pharmaceutical product for prophylactic and therapeutic use in animals and humans. Furthermore, these multi-drug formulations can reduce the overall cost and doses of mono-drug therapy. However, there are few successful combination therapies from the perspectives of diagnostics, therapeutics, and prophylactics in humans and animals. Therapeutic approaches for diseases associated with inflammation by targeting inflammatory pathways are somewhat limited. Furthermore, the approved medications are associated with several adverse effects, drug-drug interactions, and several contraindications. Hence, there is a need to develop a novel anti-inflammatory product (synthetic or herbal/natural product) with potent pharmacological effects, appropriate ADME profile (absorption, distribution, metabolism and elimination), and with minimal adverse effects. The most common animal species used for testing a product prior to human use are monkeys, dogs and rabbits. The *in vivo* results generated from rabbits are similar to the pharmacokinetic studies in humans because of phylogenetic similarity. Hence, rabbit has been used as an animal model for validating the pharmacokinetic effects of synthetic or herbal/natural products (Thomas et al., 2012). Consequently, in our study we investigated the efficiency of *Piper longum* on β-boswellic acid’s absorption profile using rabbit as an animal model.

*Piper longum,* or long pepper, is cultivated for its fruit and typically used as a spice in Asian countries as China, Indonesia and India (Kuete et al., 2013). Its usage extends well beyond culinary purposes, especially in Ayurvedic treatments, or traditional and holistic Indian and Chinese medicine. Ancient medicines have long used the pepper for migraines, strep throat, and indigestion (Gorgani et al., 2017). Piperine exhibited antioxidant (Vijayakumar et al., 2004), anti-cancer (Pradeep and Kuttan, 2002), antimycobacterial (Philipova et al., 2018), antiparasitic (Soutar et al., 2017), antiplatelet, (Iwashita et al., 2007), analgesic (Vedhanayaki, et al., 2003), antidepressant (Lee et al., 2008), and anti-inflammatory activities (Darshan and Doreswamy, 2004). The anti-inflammatory property of piperine is of specific interest due to the variety of inflammatory diseases, such as atherosclerosis, heart failure, pancreatitis, endometritis, and rheumatoid arthritis. Piperine was also effective in reducing acute inflammation, as seen by its ability to suppress edema, cotton pellet-induced granuloma (Mujumdar et al., 1990; Murunikkara et al., 2012). Furthermore, piperine dose-dependently inhibited production of interleukin-6 and prostaglandin E2 in IL 1β-stimulated fibroblast-like synoviocytes from patients with rheumatoid arthritis (Bang et al., 2009). Similar results were observed in a separate study investigating the effects of piperine revealing a reduction in the expression of TNF-α, IL-1β, and IL-6 and an increase in the expression of IL-10 (Zhai et al., 2016).

Several approaches to increase the bioavailability of boswellic extract have been investigated. For instance, the micellar delivery form of Boswellia extract and a soy lecithin formulation of boswellic extract gum resin improved systemic availability and tissue distribution. Our study utilized a novel approach to combine piperine and β-boswellic acid rich fraction to improve the pharmacokinetic properties of boswellic acid. Moreover, Piperine also drew some recent attention due to its ability to increase bioavailability of certain drugs. For example, in the case of Risorine (contains rifampicin-200 mg, isoniazid-300 mg, and piperine-10 mg), bioavailability of rifampicin was increased by 60% by piperine. Increased bioavailability allows for reductions in dosage, cost, and toxicity (Atal and Bedi, 2010). Multiple mechanisms have been proposed for the mechanism by which the piperine increases bioavailability. Hence, Piperine promotes gastrointestinal absorption by increasing secretion of bile acids (Sharma et al., 2005), up surging the gastrointestinal blood flow (Johri and Zutshi, 1992), inhibiting efflux pumps (Bhardwaj et al., 2002) and reducing the metabolism by inhibition of multiple enzymes as UDP-glucose dehydrogenase (Singh et al., 1986), cytochrome P450 (Volak et al., 2008) and different oxygenases (Sambaiah and Srinivasan, 1989). Extracts usually consist of mixture of compounds, and it is unreasonable to attribute certain effects or phenomena to one compound only. However, many studies have observed that piperine is the major alkaloid compound in the *Piper longum* extract, and it is responsible for many bioactive properties including bioenhancement of other bioactive compounds (Natarajan et al., 2006; Atal and Bedi, 2010; Liu et al., 2011; Kumar et al., 2011). Moreover, a study has been conducted using LC-MS to quantify the major five alkaloids in *Piper longum* extract, (piperine (PPR), piperlonguminine (PPL), Da,b-dihydropiperlonguminine (DPPL), piperanine (PPRA) and pellitorine (PLTR)), and confirmed that piperine is the major constituent among them (Liu et al., 2015). In our study, we observed a similar phenomenon by which *Piper longum* extract acted as a bioenhancer, by improving the bioavailability of β-boswellic acid in rabbits; and this finding is in agreement with the results from other studies (Atal and Bedi, 2010; Kesarwani and Gupta, 2013).

A body of growing reports and research has recently presented some evidence on the role of Boswellia species in several diseases due to their potent anti-inflammatory effects (Beheshti and Aghaie, 2016; Iram et al., 2017). However, the low bioavailability of α-boswellic acid, β-boswellic acid, AKBA and KBA, attributed to their metabolic instability *in vivo* by phase-1 CYP3A4 enzyme of the hepatic cytochrome P450 family caused limitation in their clinical benefits (Krüger et al., 2008; Cui et al., 2016). The hepatic cytochrome P450 family regulates metabolism of several drugs and other compounds by either inducing or inhibiting the metabolism. Thus, modulation of the hepatic metabolic pathway can likely manipulate the pharmacokinetics of β-boswellic acid and improve their overall bioavailability and efficacy. Various studies have shown that piperine inhibit the major hepatic human drug metabolizing enzyme CYP3A4 especially if these drugs are taken by oral administration (Bhardwaj et al., 2002; Singh and Duggal, 2009) For instance, the co-administration of piperine and docetaxel significantly increased the bioavailability of docetaxel and subsequently improved its anti-cancer effect in a xenograft model of human castration-resistant prostate cancer (Makhov et al., 2012).

The QikProp prediction tool clearly shows that piperine, β-boswellic acid, AKBA, and KBA possess favorable oral drug-like properties with respects to SASA parameters, absorption, metabolism, distribution, and clearance. Taken together, our current *in vivo* and pharmacodynamic/pharmacokinetic modelling work validated the benefit of *Piper* combination usage with β-boswellic acid. This study should shed some promises towards developing novel drug formulations in combating various inflammatory disorders. Taken together, our current findings strongly suggest that piperine can be used as a potential bioavailability enhancer with AKBA and KBA.

## Data Availability Statement

The original contributions presented in the study are included in the article/Supplementary Material, further inquiries can be directed to the corresponding authors.

## Ethics Statement

The animal study was reviewed and approved by institution’s animal ethical committee PCP/IAEC/009/2008.

## Author Contributions

KV, and MD planned and funded the project. The experiments were carried by MG, SR, MA, MM, AF, MA, NK, JR, JB and FS. KV, MD, TM and JR analyzed the data. JR, MD, MG, and SR were involved in preparation and editing of manuscript.

## Conflict of Interest

The authors declare that the research was conducted in the absence of any commercial or financial relationships that could be construed as a potential conflict of interest.
